# Correlation analysis of risk factors for cervical lymphatic metastasis in papillary thyroid carcinoma

**DOI:** 10.1186/s13000-024-01440-1

**Published:** 2024-01-13

**Authors:** Haoying Sun, Xueyu Zhao, Xin Wang, Jinzhu Ma, Ming Liu

**Affiliations:** 1 Department of Thyroid and Breast Surgery, The Affiliated Hospital of Inner Mongolia Medical University, No. 1 Tongdaobei Street, Hohhot, Inner Mongolia 010050 China; 2 Inner Mongolia Cancer Hospital, Hohhot, China

**Keywords:** Papillary thyroid carcinoma, Cervical lymph node metastasis, Relevant risk factors, BRAF gene mutation

## Abstract

**Objective:**

This study aims to identify and analyze the risk factors associated with Cervical Lymph Node Metastasis (CNM) in Papillary Thyroid Carcinoma (PTC) patients.

**Methods:**

We conducted a retrospective study involving the clinicopathological data of 2384 PTC patients admitted to our hospital between January 2016 and December 2020. All relevant data were statistically processed and analyzed.

**Results:**

The related risk factors for Central Lymph Node Metastasis (CLNM) were gender (male), age (≤ 30 years old), tumor lesion size (> 0.855 cm), and multifocal tumor foci. The ROC curve revealed that the critical value for predicting CLNM based on tumor lesion size was 0.855 (sensitivity = 57.9%, specificity = 69%, AUC = 0.269, and *P* < 0.05). Lateral Lymph Node Metastasis (LLNM) was positively correlated with tumor diameter. Specifically, the LLNM rate increased with the tumor diameter. LLNM occurrence was significantly higher in zones II, III, and IV than in zones I and V. Although the BRAF gene mutation detection assay has certain clinical benefits in diagnosing PTC and LLNM, no statistically significant difference was found in its relationship with central and lateral neck lymph node metastases (*P* = 0.741).

**Conclusion:**

Our findings revealed that CLNM is associated with gender (male), age (≤ 30 years old), tumor lesion size (> 0.855 cm), and multiple tumor lesions in PTC patients. Central Lymph Node Dissection (CLND) is recommended for patients with these risk factors. On the other hand, preoperative ultrasound examination, fine-needle pathological examination, and genetic testing should be used to determine whether Lateral Cervical Lymph Node Dissection (LLND) is needed.

## Introduction

Thyroid cancer accounts for about 1% of all malignant tumors in humans and around 33% of head and neck malignant tumors [1]. Furthermore, Papillary Thyroid Carcinoma (PTC) accounts for approximately 80–90% of thyroid malignant tumors. The global incidence of thyroid cancer has increased rapidly in recent years, at a rate of 4.5–6.6% annually [2]. Although most PTC patients have a good prognosis, it is noteworthy that 20–90% of PTC patients can develop Lymph Node Metastasis (LNM) [3]. In addition to increasing the risk of local recurrence, LNM can lower the Disease-Free Survival (DFS) rate of PTC patients. Furthermore, LNM may lead to secondary surgery or radiation iodine therapy, affecting patients’ Quality of Life (QoL) [4]. The PTC LNM often occurs in the central region at first, followed by the lateral cervical region, and finally the mediastinal lymph nodes [5]. However, it is noteworthy that metastatic progression does not strictly adhere to these steps, as some stages could be skipped. Therefore, a comprehensive, rational, and appropriate initial surgery can reduce the risk of postoperative recurrence and the possibility of secondary surgery. Herein, we retrospectively analyzed the clinicopathological data of 2384 PTC patients, focusing on the risk factors of Central Lymph Node Metastasis (CLNM) and Lateral Lymph Node Metastasis (LLNM) in PTC patients. We also explored the surgical methods for treating PTC.

## Materials and methods

### General information

The Medical Ethics Committee of Inner Mongolia Medical University Affiliated Hospital approved our research plan. Our hospital admitted 2709 thyroid malignant tumor patients from January 2016 to December 2020. Among them, 2384 [88.0%, 353 males (14.8%) and 2031 females (85.2%), age range = 15–83 years, average age = 46.41 ± 10.23 years] were PTC patients. Preoperative ultrasound examination of the thyroid and neck lymph nodes, chest X-ray, and chest CT were performed on all patients, with some undergoing neck CT examination. Preoperative fine-needle biopsy was performed on 210 patients (10%), of which 179 were diagnosed with PTC and LNM. The diagnosis was confirmed through intraoperative freezing and postoperative pathological examination, which yielded a positive rate of 85%. All patients underwent an intraoperative frozen section pathological examination during surgery and a routine postoperative pathological examination. The exclusion criteria were as follows: ① Patients with a previous history of malignant tumors; ② Patients undergoing the present operation as a secondary surgery; ③ Patients that were pathologically diagnosed with non-papillary carcinoma; ④ Patients with upper mediastinal LNM or distant metastasis; ⑤ Patients who underwent non-radical surgery; and ⑥ Patients who did not undergo follow-up examination six months post-surgery.

### Surgical methods

All PTC patients included herein underwent radical thyroidectomy. Among them, 1829 (76.7%) and 555 (23.3%) patients underwent unilateral Central Lymph Node Dissection (CLND) and bilateral CLND, respectively. Preoperative ultrasound examination revealed enlarged lymph nodes in the lateral neck, while Fine-Needle Aspiration (FNA) biopsy showed LNM in 38 cases (1.6%), prompting the need for Lateral Cervical Lymph Node Dissection (LLND).

### Related data analysis

Among the 1829 patients that underwent unilateral CLND, 887 had Central Lymph Node Metastasis (CLNM), with a metastasis rate of 48.5%. On the other hand, among the 555 patients who underwent bilateral CLND, 287 had CLNM, with a metastasis rate of 51.5%. Furthermore, 38 patients underwent lateral neck lymph node dissection, of which 32 cases were confirmed through postoperative pathological examination to have LLNM, with a metastasis rate of 1.3%. Gender, age, tumor size on histology, and multifocal tumor nature (single or multiple lesions) were subjected to univariate analysis to determine if they are linked to a higher risk of central and lateral neck lymph node metastases in 2384 PTC patients (Tables 1 and 2), and BRAF gene testing was performed on 85 patients (Table 3).

### Follow up

A follow-up rate of 91.0% was achieved, with 2169 of the 2384 PTC patients completing the full follow-up regimen, while 25 patients were lost. The follow-up period was set for 12–72 months up until December 31, 2021. Among the patients who did not undergo LLND, 47 (2.0%) experienced lateral neck metastasis post-surgery. There were 4 (0.2%) and 2 (0.1%) cases of lung metastasis and bone metastasis, respectively, and no deaths were reported.

**Table 1:** Univariate analysis of CLNM in 2384 PTC patients who underwent CLND [n (%)]

Influencing factor		Number	Is the central lymph node metastatic		OR	95%CI	P
			Yes	No			
Gender							
Male		353	236(66.9)	117(33.1)	0.181	1.141–2.232	< 0.001
Female		2031	938(46.2)	1093(53.8)	1.000		
Age (years)							< 0.001
	≤ 30	151	127(84.1)	24(15.9)	5.926	3.894–9.021	< 0.001
	30–55	1694	781(46.1)	913(53.9)	1.216	0.995–1.485	0.06
	>55	539	266(49.4)	273(50.6)	1.000		
Lesion (cm)							< 0.001
	≤ 0.5	1015	356(35.1)	659(64.9)	1.000		
	0.5-1	905	454(50.2)	451(49.8)	1.863	1.551–2.238	< 0.001
	1–2	347	258(74.4)	89(25.6)	5.366	4.083–7.052	< 0.001
	2–4	107	97(90.7)	10(9.3)	7.956	4.609–11.757	< 0.001
	>4	10	9(90.0)	1(10.0)	6.660	1.510-12.599	0.008
Multifocal	Single stove	1573	536(34.1)	1137(72.3)	1.000		
	Multifocal	811	638(78.7)	173(21.3)	7.823	6.425–9.525	< 0.001

**Table 2:** Univariate analysis of LLNM in 38 PTC patients who underwent LLND [n (%)]

Influence factor		Number of cases	Is the lateral lymph node metastatic		OR	95%CI	P
			Yes	No			
Gender							
Male		12	10(83.3)	2(16.7)	0.909	0.142–5.809	0.920
Female		26	22(84.6)	4(15.4)	1.000		
Age (years)							0.739
	≤ 30	8	7(87.5)	1(12.5)	1.750	0.084–36.28	0.718
	30–55	25	21(84.0)	4(16.0)	1.000	0.091–11.028	0.978
	>55	5	4(80.0)	1(20.0)	1.000		
Lesion (cm)							0.955
	≤ 0.5	4	3(75.0)	1(25.0)	1.000		
	0.5-1	9	7(77.8)	2(22.2)	1.167	0.109–25.433	0.913
	1–2	12	10(83.3)	2(16.7)	1.667	0.109–25.433	0.713
	2–4	10	9(90.0)	1(10.0)	3.000	0.140-64.262	0.482
	>4	3	3(100.0)	0(0)			
Multifocal	Single stove	15	12(80.0)	3(20.0)	1.000		
	Multifocal	23	20(87.0)	3(13.0)	0.600	0.104–3.463	0.568
Number of lymph node metastases in the central region (number)							0.374
	0	13	10(76.9)	3(23.1)	0.208	0.019–2.290	0.200
	≤ 3	8	6(75.0)	2(25.0)	0.188	0.014–2.468	0.203
	>3	17	16(94.1)	1(5.9)	1.000		
Distant metastasis		1	1(100)	0(0)			

**Table 3:** Analysis of the correlation between gene mutations and CLNM in patients undergoing BRAF gene testing [n (%)]

Group	Total	Lymph node metastasis	No lymph node metastasis	χ^2^	P
BRAF mutation	79	43 (54.4)	36 (45.6)	0.109	0.741
BRAF not mutated	17	10 (58.8)	7 (41.2)		

### Statistical methods

All statistical analyses were performed using SPSS 26.0 software. Counting data were expressed as percentages. The *χ*^*2−*^test or Fisher’s exact probability method was used for component comparisons. Binary logistic regression analysis was performed to analyze the relevant risk factors using PTC neck lymph node metastasis as a variable factor (0-no, 1-yes). We used ROC curves to determine the critical value for predicting CLNM based on the size of tumor lesions. Inspection level *α* = 0.05.

## Results

### Univariate analysis of factors related to CLNM

Univariate analysis revealed a significant correlation between CLNM and gender, age, lesion size, and multifocal characteristics in PTC patients (*P* < 0.05).

### Multivariate analysis of factors related to CLNM

**Fig. 1:**
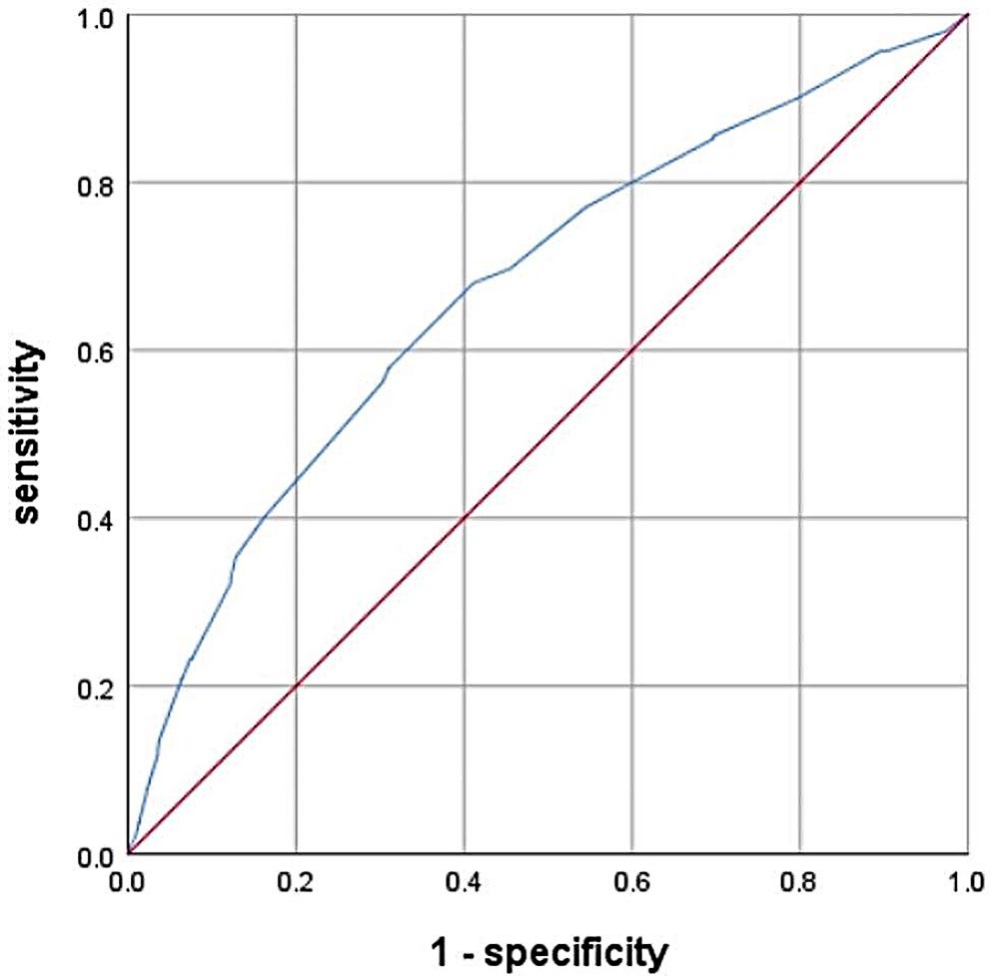
ROC curve analysis predicts CLNM based on tumor lesion size. The ROC results show that 0.855 is the critical tumor lesion size value and the best point for predicting CLNM. The sensitivity, specificity, AUC, and 95%CI at this value are 57.9%, 69%, 0.269, and 0.639–0.707, respectively

Herein, we constructed a multivariate logistic regression equation by incorporating gender, age, lesion size, and the multifocal tumor nature. According to the results, the risk of CLNM was significantly higher in males than females (*OR*:5.294,95% *CI*: 3.768–7.438, *P* < 0.05). On the other hand, the risk of CLNM increased with decreasing age and increased with increasing lesion size and number of multifocal lesions (*OR*:3.188, 95% *CI*: 1.963–5.176, *P* < 0.05) (Table 4). We created ROC curves for 2384 patients undergoing CLND to further investigate the relationship between CLNM and tumor lesion size. We determined that the critical value for predicting tumor lesion size was 0.855. The AUC was 0.269, with sensitivity and specificity values of 57.9% and 69%, respectively (*P* < 0.05) (Fig. 1). The CLNM rates of patients with BRAF gene mutations and those without BRAF gene mutations were 54.4% and 45.6%, respectively. No statistically significant difference was found in the transfer rate between the two groups (*P* = 0.741) (Table 3).

**Table 4:** Multivariate analysis of factors related to CLNM in 2384 PTC patients [n (%)]

Influencing factors	Group	Number of cases	Is the central lymph node metastatic		OR	OR95% CI	P-value
			Yes	No			
Gender							
	Male	353	236 (66.9)	117 (33.1)	5.294	3.768–7.438	< 0.001
	Female	2031	938 (46.2)	1093 (53.8)	1.000		
Age (years)							< 0.001
	≤ 30	151	127 (84.1)	24 (15.9)	3.188	1.963–5.176	< 0.001
	30–55	1694	781 (46.1)	913 (53.9)	1.398	0.905–1.945	0.078
	>55	539	266 (49.4)	273 (50.6)	1.000		
Lesion size (cm)							< 0.001
	≤ 0.5	1015	356 (35.1)	659 (64.9)	1		
	0.5- 1	905	454 (50.2)	451 (49.8)	2.924	2.227–3.841	< 0.001
	1–2	347	258 (74.4)	89 (25.6)	4.000	2.801–5.711	< 0.001
	2–4	107	97 (90.7)	10 (9.3)	6.740	2.801–10.862	< 0.001
	>4	10	9 (90.0)	1 (10.0)	5.168	1.218–21.930	0.026
Multifocal	Single	1573	536 (34.1)	1137 (72.3)	1.000		
	Many	811	638 (78.7)	173 (21.3)	0.667	0.515–0.863	0.002

### Analysis of factors related factors to LLNM

Analysis of 38 PTC patients who underwent LLND revealed that the size and number of lesions, as well as the number of CLNMs, were correlated with LLNM (Table 2). We also compared LNM in different lateral neck regions (Table 5). The LNM rate was higher in zones II, III, and IV than in zones I and V. However, the groups studied had relatively fewer cases, necessitating additional in-depth analysis and research with more cases for each category.

**Table 5:** Analysis of lateral CNMin different regions for the 38 patients who underwent routine LLND [n (%)]

Tumor regions	Number of cases (person)	Is the lateral lymph node metastatic		χ^2^	*P*
		Yes	No		
Zone I	3	0 (0.0)	3 (100.0)	1.357	0.852
Zone II	22	13 (59.1)	9 (40.9)		
Zone III	29	19 (65.5)	10 (34.5)		
Zone IV	27	17 (63.0)	10 (37.0)		
Zone V	10	3 (30.0)	7 (70.0)		

## Discussion

According to research, PTC, the most common type of thyroid cancer, has an excellent 10-year Survival Rate (SR) of over 90% [6]. Nonetheless, LNM occurs in 20–90% of PTC cases and is generally considered the primary cause of PTC local recurrence. It has been reported that secondary surgery post-recurrence increases the difficulty of postoperative care, reduces patients’ QoL, and affects patients’ SR [7]. Lymph node dissection during PTC surgery increases the risk of iatrogenic complications. Currently, a great controversy remains over the extent of preventive CLND and therapeutic LLND. Balancing treatment methods, avoiding overtreatment of low-risk patients, and identifying patients with more severe conditions or at higher risk of injury (for whom more active treatment methods are needed) are some of the challenges currently faced by Doctors taking care of these patients. Therefore, understanding the nature and risk factors of Cervical Lymph Node Metastases (CNMs) in PTC patients is critical in guiding CLND.

Consistent with other research findings [8, 9], we found an increased risk of CLNM in male patients in this study. This outcome indicates that special emphasis should be placed on evaluating LNM in male PTC patients during preoperative clinical examinations. Herein, PTC patients aged ≤ 30 years were more likely to develop CLNM. Although this finding aligns with some previous research [10, 11], it is noteworthy that some scholars [12] found that CLNM is not related to age. Furthermore, Yang [10] deduced that > 44.5 years old is the threshold for cervical lymph node skip metastasis, with patients in this age group being more prone to cervical lymph node skip metastasis. This study revealed that tumor size (> 0.5 cm) is a risk factor for CLNM in PTC patients, with a tumor size of 0.855 cm as the critical value for predicting CLNM according to the ROC curve. In PTC research, scholars have consistently reported that tumor size is an essential factor in predicting CNM, but with different thresholds. However, it is generally believed that the larger the tumor lesion, the higher the CNM risk [13, 14]. Consistent with other research results [15], multifocal tumor foci are also a risk factor for CNM in PTC patients. Furthermore, Liu [11] found that the extension and growth of tumors outside the thyroid gland is a risk factor for CLNM, potentially because the tumor cells invading perithyroidal soft tissue are more likely to metastasize along the rich lymphatic tissue to the surrounding lymph nodes, resulting in LNM. No statistically significant relationship was found between BRAF gene mutations and CLNM. In this regard, BRAF gene mutations are not found to be a risk factor for CLNM.

Although many studies have been conducted on the characteristics and risk factors of LLNM, the findings are highly controversial. Zhang et al. [16] reported that tumors extending outward from the thyroid gland, bilateral lobe tumors, and CLNM are risk factors for LLNM. On the other hand, Niel et al. [17] reported that tumors located at the upper pole, CLNM, and tumors > 1.5 cm in size are risk factors for LLNM. Furthermore, Liu agrees that CLNM is a risk factor for LLNM and that LLND should be conducted more actively when the number of CLNMs is more than three. Contrastingly, a previous study [14] reported that CLNM is not a risk factor for LLNM. This study found that the rate of lateral CNM increased with the increase in tumor size, but the difference was not statistically significant. There is currently no consensus on the scope of lateral neck lymph node dissection, and a controversy remains over whether to routinely clean lymph nodes in Zone V [18, 19]. Here, we found that LLNM mainly occurs in zones II, III, and IV, with less occurrence in regions I and V. It may be appropriate to not clean the lymph nodes in Zones I and V for PTC patients with low-risk factors.

Dr.Ozgur’s team review of the thyroid gland confirms that the thyroid gland has no defined anatomical fibrous capsule,but rather perithyroidal soft tissue [20]. Furthermore, some scholars highlighted the importance of tumor location in the perithyroidal soft tissue, discovering that tumor invasion of the soft tissue could increase the risk of tumor recurrence and death [21]. Wang et al. [22] discovered that PTC invasion and breakthrough of the perithyroidal soft tissue or posterior dorsal soft tissue increases the likelihood of tumor invasion into lymphatic vessels and the risk of CNM.

According to recent research, the V600E mutation of BRAF (v raf murine sarcoma viral oncogene homolog B1) is the most common and critical genetic event in PTC occurrence. The BRAF V600E mutation is solely found in PTC and PTC-derived undifferentiated cancers, and is absent in normal thyroid tissue, thyroid follicles, and other types of thyroid tumors [23]. Numerous studies reported that this mutation is associated with commonly known clinicopathological features of PTC that predict tumor progression and recurrence, such as advanced age, extrathyroidal invasion, LNM, and advanced tumor stages. Additionally, the direct association between the BRAFV600E mutation and clinical PTC progression, recurrence, and treatment failure has been confirmed. Herein, 79 of the 85 PTC patients who underwent BRAF gene testing were found to have BRAF gene mutations, with a mutation rate of 93%. One study indentified several molecular and histopathologic features that correlate with more behavior of Thyroid papillary microcarcinoma(TPMC),such as BARF mutation status, subcapsular location, peri-and intratumoral fibrosis, and multifocality, and provided a practical and simple scoring system to evaluate the clinical behavior of this common type of thyroid cancer. The scoring system relies on BRAF mutation status and three histopathological features to assign tumors into three risk categories. The absence of either of these factors cannot be accurately classified [24]. However, our BRAF gene testing sample size was relatively small and incomplete histopathological features information, necessitating additional research with larger samples to obtain more accurate conclusions. Furthermore, in a previous study, 13 of the PTC patients who underwent BRAF testing and showed no mutations were complicated with Hashimoto’s Thyroiditis (HT). Some studies discovered that the inflammatory process of HT exerts a protective effect on PTC [10]. Many patients with PTC + HT were clinically diagnosed with enlarged cervical lymph nodes [25], which posed more difficulties for preoperative color Doppler ultrasound for determining LNM, leading to more lymph node clearance and complications. As a result, accurately identifying the risk factors for CLNM and determining the need for neck lymph node dissection is even more critical for PTC patients with HT.

## Summary

Our study findings can be summarized in five key points. First, male patients, patients aged ≤ 30 years, and those with a tumor lesion size > 0.855 cm should undergo preventive CLND. Second, LLNM presence should be confirmed through color ultrasound examination and fine-needle biopsy before LLND. Third, the lymph node cleaning range should include Zones II, III, and IV, whereas lymph nodes in Zones I and V can be cleaned as appropriate. Fourth, the appropriate surgical method and whether lateral neck lymph node dissection is necessary could be determined through preoperative puncturing of tumor lesions and assessment of enlarged lateral neck lymph nodes. Despite the above-mentioned insightful findings, this study has some shortcomings. Particularly, clinical examination results, imaging features, and tumor location were not examined. Consequently, additional research is required to further explore the relevant risk factors for CNM in PTC patients.

## Data Availability

The datasets generated and analysed during the current study are not publicly available because internal statistical data of the research unit and has not been uploaded to the database, but are available from the corresponding author on reasonable request.
